# Recombination and phylogenetic inference

**DOI:** 10.1093/evolinnean/kzaf016

**Published:** 2025-08-21

**Authors:** Bruce Rannala

**Affiliations:** Department of Evolution and Ecology, University of California Davis, Davis, CA 95616, United States

**Keywords:** recombination, species tree inference, coalescent, ancestral recombination graph

## Abstract

I explore the problem of inferring phylogenetic trees in the presence of recombination. Two widely used approaches are considered: concatenation methods assume all loci have one underlying gene tree; and species tree inference methods assume one gene tree underlies each locus (no intralocus recombination) and loci have independent gene trees (high interlocus recombination). The impact of recombination is different under these two approaches. Three strategies for addressing the impacts of recombination are considered: (i) studies of the statistical robustness of phylogenetic inference methods when recombination occurs and is not accounted for (if impacts are minimal, recombination can be safely ignored); (ii) methods that accommodate recombination by identifying recombinant regions to either eliminate recombinant loci (to reduce intralocus recombination) or to choose loci that are separated by multiple recombinations (to increase interlocus recombination); and (iii) methods for phylogenetic inference that aim to accommodate recombination by inferring breakpoints between regions of sequences with different gene trees or allow varying topology along a sequence. I conclude that recombination is likely to be more detrimental for concatenation methods, having little impact on topology or divergence time estimates for species tree inference methods. Recombination detection may not be necessary when performing species tree inference, and eliminating recombinant loci may bias parameter estimates. Methods allowing gene trees to vary across the genome still lack theory-based criteria for combining inferred gene trees to estimate a species tree; this could in principle be done using a multispecies coalescent model with recombination but is a considerable technical challenge.

## INTRODUCTION

Early approaches to molecular systematics focused on inferring phylogenetic relationships using a single gene sequence sampled from each species (Hillis *et al.* 1996). However, if recombination occurs it will potentially create differences of gene tree topologies and branch lengths among regions of the genome. With multilocus data (or complete genome sequences) from organisms that undergo recombination it therefore becomes essential to consider the impact of recombination on inferred phylogenetic trees (Martin *et al.* 2011). Over the last several decades much research by both population geneticists and phylogeneticists has focused on the phylogenetic impact of recombination. The problem of recombination in phylogenetics has been approached from three distinct angles: (i) analyses of the robustness of different phylogenetic inference methods to the effects of recombination; (ii) accommodation of recombination in phylogenetic inferences by detecting (and excluding) recombinant sequences; or (iii) the development of new methods that aim to incorporate recombination.

Although recombination is the mechanism that allows gene trees to change across the genome, the population coalescent process is fundamental in determining the probability distribution of gene trees underlying segments of the genome. When considering samples from populations or species the distribution of gene trees depends on concurrent coalescent processes within different populations, referred to as the multispecies coalescent (MSC). During the last decade interest has shifted from inferring individual gene trees to species tree inference using multilocus data with variable gene trees generated under the MSC (Edwards 2009). The MSC is an extension of the coalescent theory (Kingman 1982) to multiple populations or species (reviewed in Rannala *et al.* 2020). MSC-based phylogenetic inference methods currently assume no intralocus recombination (each locus has a single underlying gene tree) and free interlocus recombination (loci have independent gene trees) rather than explicitly modelling recombination (due to the computational challenges). Instead, developers of those programs typically advise users to choose loci comprising short widely distributed sequences, minimizing the intralocus and maximizing the interlocus probabilities of recombination. One can easily simulate samples under an MSC with recombination to evaluate the effects of recombination on such methods. The standard framework used for most recent studies of phylogenetic robustness to recombination is therefore the MSC with recombination (see section on robustness of species tree inference). Recombination is a mechanism that generates gene tree variation across a sequence and one can also study the phylogenetic effects of gene tree variation among sites without specifying a mechanism; phylogenetic models that allow gene trees to vary independently among sites (mixture models) are therefore relevant, despite not explicitly modelling recombination, and are examined here also. I begin by briefly describing the coalescent with recombination and the ancestral recombination graph (ARG; Hudson and Kaplan 1985, Griffiths and Marjoram 1996) for a single population which is fundamental to understanding the parameters of the MSC–ARG.

## The coalescent with recombination

Here we outline the coalescent process with recombination (Hudson 1983) assuming a single panmictic population, defining the parameters that will be referenced in subsequent sections. We consider a random sample of chromosomes from a neutral Fisher–Wright (FW) model in the limit of a large population and small sample. Although the FW model assumes discrete nonoverlapping generations, the coalescent with recombination approximates an FW model (with recombination) as a continuous-time Markov chain (CTMC). Going backwards in time this is a birth–death process. A recombination event is a birth event creating two chromosomes, each partially ancestral to the sample, and a coalescence event is a death collapsing two chromosomes into a common ancestral chromosome.

**Figure 1.**
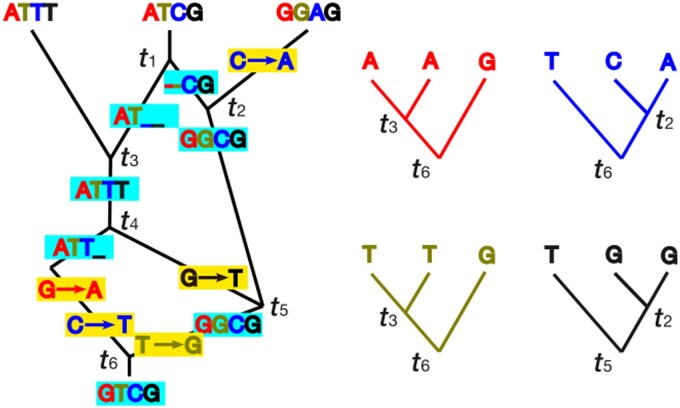
The relationship between the ancestral recombination graph (ARG) (at left) and site-specific gene trees (at right) is illustrated for a sample of 4-bp sequences from each of three chromosomes. From the top to the bottom of figure time increases from the present into the past. The lines (edges) represent chromosomal lineages through time. Edge intersections (vertices) with two descendant edges (above them) represent coalescent events and vertices with two ancestral edges (below them) represent recombination between adjacent sites. Nucleotides are coloured by position (1: red, 2: green, 3: blue, 4: black) and gene trees (at right) are coloured according to the site that they represent. Ancestral mutations are indicated by yellow boxes and ancestral sequences are represented by blue boxes with an underscore representing sites/segments that are unknown (not ancestral to the sample) because of recombination. The variable $t_{i}$ represents the time of an adjacent recombination or coalescence event. In this example, there are three distinct gene trees (sites 1 and 2 share the same gene tree and coalescent times).

Define $N_{e}$ to be the diploid effective population size, *n* to be the sample size, and *r* to be the expected number of recombinations per base per generation. Here we also include a DNA mutation process with mutations occurring at rate μ per base per generation. Consider $n_{t}$ chromosomes ancestral to the sample at a time *t* generations in the past. Three types of events occur: coalescence events occur with rate


$$\left(\begin{array}{c} n_{t}\\ 2 \end{array}\right)\frac{1}{2N_{e}},$$

recombination events occur with per-chromosome rate *rs*, and mutation events occur with per-chromosome rate μs, where *s* is the total length of DNA (number of sites) ancestral to the sample on the set of chromosomes from which the sampled chromosomes descend. The coalescent process was originally formulated using a diffusion timescale of $2N_{e}$ generations. On a diffusion timescale with units of $2N_{e}$ generations the canonical rates are obtained from the above rates by multiplying each by $2N_{e}$. For example, the rate of coalescence is then


$$\left(\begin{array}{c} n_{t}\\ 2 \end{array}\right),$$

the rate of recombination is $2N_{e}rs$, often instead written as (ρ/2)s where $\rho =4N_{e}r$, and the rate of mutation is $2N_{e}\mu s$ often instead written as (θ/2)s where $\theta =4N_{e}\mu$. The waiting times between coalescence, recombination, and mutation events in this model are exponential random variables whose parameters are determined by the rates defined above. An ARG representing the history of a sample of three DNA sequences, each comprising four sites, is shown in Figure 1. For more details on these standard results from coalescent theory see Wakeley (2009). When inferring phylogenetic relationships of populations or species the coalescent process operates within each population (species) including the ancestral populations and is referred to as the MSC (reviewed in Rannala *et al.* 2020). One can also allow recombination and migration in an MSC (Hudson 2002).

## PHYLOGENETIC ROBUSTNESS TO RECOMBINATION

Current approaches to phylogenetic inference can be broadly classified as either ‘universal’ gene tree or species tree inference methods. The universal gene tree inference methods assume that a single gene tree underlies all the sequence data being analysed and seek to infer that tree. The species tree inference methods (as noted above) assume that a shared species tree exists for all sites (and loci) but that the gene tree at any site is an independent random variable from the probability density of gene trees under the MSC, which is determined by parameters such as the species tree, species divergence times, and effective population sizes of contemporary and ancestral populations. The effects of recombination on these two inference approaches are very different and have thus been the subject of different studies with different designs. Simulated sequences have often been used to study the effects of recombination because the analytical theory is typically intractable. However, related phylogenetic mixture models do allow some relevant analytical solutions (see section on modelling gene tree mixtures).

## Robustness of universal gene tree inference

We begin by considering universal gene tree inference methods; with multilocus datasets these are also known as ‘concatenation’ methods because the universal gene tree assumption justifies concatenation of all loci into a single sequence for analysis (although one might still partition the data to accommodate substitution rate variation among loci, etc.) The robustness of phylogenetic inferences of gene trees to recombination has been studied using two approaches: (i) explicit simulation of the recombination process producing gene trees underlying different sites; and (ii) analytical or simulation studies of mixture distributions with different trees underlying different sites but no mechanistic connection to recombination. We consider results obtained using both approaches.

Because the MSC operates in most populations and species, potentially causing gene trees to differ from a species tree (a process referred to as incomplete lineage sorting; ILS) (Avise 1994) one might wonder whether there is ever any justification for using concatenation? Concatenation is most appropriate in situations where the gene tree has a very high probability of matching the species tree at any site in the genome. In such cases, the homologous recombination process is irrelevant. Such conditions might occur if effective population sizes are very small and/or the intervals between population divergence events are very long and there is no introgression between species (Palumbi *et al.* 2001) but even in such cases outcomes are highly stochastic and gene trees for some loci are likely to conflict (Hudson and Turelli 2003) unless parameter combinations are extreme.

A different situation occurs with genomes not experiencing recombination, organelle genomes for example. In that case, if ILS occurs, the gene tree may not match the species tree (Hudson and Turelli 2003) but gene trees from different parts of the genome should still be identical, justifying concatenation (although not as a species tree inference method). In practice, one can never be confident that any of these conditions are met, although one can potentially test for heterogeneity of gene trees along a sequence (see section on phylogenetic mixture models) when analysing empirical datasets; this could justify concatenating the data if heterogeneity is not evident. Instead of assuming that such ideal conditions are met, or testing empirical datasets for topology heterogeneity, one could instead ask whether phylogenetic analysis using concatenation is robust to some amount of recombination and gene tree variation. The simulation studies and theoretical analyses considered next focus on that question.

## Modelling gene tree recombination

Posada and Crandall (2002) studied the effect of recombination on phylogeny estimation using maximum parsimony, minimum evolution, and maximum likelihood. Their simulations assumed one sequence was sampled from each species and a single recombination event occurred that resulted in either a reciprocal or nonreciprocal exchange. This approach implicitly assumes gene flow between the species experiencing the recombination event but the single breakpoint model does not fit expectations under a population genetic model. In a population genetic model of a sexual species the introgressed portion on a chromosome is broken up and reduced by recombination over time so that its total length is a function of the time since introgression (Pool and Nielsen 2009). The authors reason that because sites are assumed to evolve independently under the phylogenetic inference model the actual number of recombinations is irrelevant to the phylogenetic likelihood and only the proportion of sites from each tree matters. This is correct, but one still expects the proportion of introgressed sites to be a function of the age of the introgression event which is not the case in their model. The authors’ model of reciprocal exchange between species also does not fit expectations under a population genetic model.

Posada and Crandall (2002) modelled the effects of recombination on topologies by switching tip labels between trees underlying segments to the left and right of the recombination breakpoint. They distinguished between ‘ancient’ exchanges involving tips connected through long branches, ‘divergent’ exchanges involving tips connected through many edges, and ‘close’ exchanges involving tips connected through few edges and short branches. Recombinations were placed to allow 50, 75, 90 or 100% (no recombination) of sites to evolve on a particular topology. Despite the somewhat tenuous connection of this simulation model to biological recombination processes the results may still provide some general insights about the effects on phylogenetic inferences of conflicting topologies with different amounts of sequence support. Since there are just two trees (a left and a right tree) in this case one can evaluate the phylogenetic support for either of these trees (or possibly a third alternative) under the different inference methods. They found that ‘divergent’ exchanges with 50% of sites evolving under each topology most often led to confounding (a topology that did not match either the left or right topology) and in other cases the topology underlying the majority of sites was recovered.

Leaché and Rannala (2011) compared the statistical performance of different species tree inference methods when sequences are generated under an MSC model with loci that are completely unlinked. This should be the worst-case scenario for a concatenation method. Indeed, they found that in difficult ranges of the parameter space with asymmetrical species trees, large θ, and short branches the accuracies of Bayesian or parsimony analysis using concatenated sequences approached 10–20% whereas a Bayesian MSC method (BEST) (Liu 2008) had accuracies of 50% or higher. Conversely, with symmetric trees, small θ, and large branches all the methods, including concatenation, approached 100% accuracy. This illustrates that there are regions of parameter space in which concatenation may perform well, but MSC-based species tree inference methods perform as well in those parameter domains but still outperform concatenation elsewhere, providing few reasons for using concatenation analyses in analysing data from species undergoing recombination other than computation efficiency.

## Modelling gene tree mixtures

Parametric statistical models in phylogenetics typically assume that individual sites in a DNA sequence are independent and identically distributed (iid) random variables whose probability distribution function (PDF) is specified by the gene tree (with branch lengths in units of expected substitutions) and the substitution model. If recombination creates variation in the gene tree along a sequence, different sites will have different PDFs and are thus no longer iid. The model can be relaxed to instead assume only that sites are exchangeable random variables. This allows the sequence to be partitioned into finite sets, with sites belonging to the same set being identically distributed and all sites being conditionally independent. In the phylogenetics literature such exchangeable models are referred to as mixture models (Yang and Rannala 2012) and partition membership is assumed to be independent of the location of a site in a sequence (although it could depend on codon position, etc). Mixture models are a general device that can be used to model variation in the DNA substitution process among sites as well as variation in gene trees (reviewed in Rannala and Yang 2008), and here we focus exclusively on mixture models of gene tree variation. The gene tree mixture model is not explicitly based on recombination and ignores the correlation of gene trees (and resulting linkage disequilibrium) at adjacent sites along a sequence that is expected with recombination. However, the mixture approach is still useful for modelling the general effects of recombination or other processes that produce changes in the gene tree among sites. It may underestimate statistical power by comparison with an explicit model of recombination, however, as it ignores potential information available from site positions in the sequence.

Wiens (1998) proposed a heuristic procedure for analysing subsets of sequence data and comparing inferred topologies to decide whether the sequence data could be combined. Analyses of simulated sequence data suggested that if gene tree differences were minor a combined analysis could improve inferences of a common tree. However, this method does not allow inferences to be combined across datasets when gene trees are very different; this is because no theoretical connection is provided between inferred gene trees and the species tree. The simulation studies of both Wiens (1998) and Posada and Crandall (2002) found that combined datasets with multiple underlying gene trees could lead to inferred gene trees that do not match any of the underlying gene trees. This implies that a concatenated analysis could infer an incorrect topology even if ILS does not cause gene tree topologies to differ from the species tree and only causes variation of gene tree branch lengths.

Mossel and Vigoda (2005) simulated datasets with an equal mixture of two five-taxon trees and examined the effects on convergence of Bayesian Markov chain Monte Carlo (MCMC) methods for phylogenetic tree inference. They found that a tree mixture slowed MCMC convergence and could create a false impression of high posterior probability for a particular tree. However, Ronquist *et al.* (2006) responded that running multiple chains with different starting points, as is commonly done in practice, would immediately reveal this problem to users and using a model of tree mixtures (rather than a misspecified model assuming a single tree) could potentially solve the mixing problems. However, they did not demonstrate that a correctly specified model with a mixture of topologies improved mixing. Thus, recombination may be a source of MCMC mixing problems in Bayesian phylogenetic analysis as well.

The effects of gene tree mixtures on phylogenetic inferences have also been explored using a simple model with two state characters and a mixture of two four-taxon gene trees that only differ in their branch lengths (Matsen and Steel 2007). The question of interest in these analyses was whether mixtures of trees exist that produce identical expected site frequencies as a single tree with a specified set of branch lengths. If that is the case then the mixture is not identifiable and cannot be distinguished from data produced under a single tree. They showed that such cases occurred under their model. However, Allman *et al.* (2011) extended their model to consider four state characters under simple substitution models and showed that the mixture model was identifiable in this state space, which is obviously more relevant for DNA sequence data. Identifiability is a minimum criterion for inference and more studies are needed to determine how much information is available to differentiate data produced under a mixture of gene trees and the loss of information due to the exchangeability assumptions of current phylogenetic models which causes potential spatial information about gene trees underlying particular sites to be ignored.

The general findings from studies of the robustness of concatenated gene tree inference suggest that mixtures of trees, if unaccounted for, can lead to phylogenetic trees that are not representative of the species trees, or even of any of the underlying gene trees. Methods have subsequently been proposed for inference in the presence of mixtures of topologies (see section on phylogenetic mixture models). Another possible solution is to use species tree inference methods based on the MSC, making explicit assumptions about the extent of linkage disequilibrium. While concatenation methods are potentially sensitive to any level of recombination (unless no ILS occurs), MSC methods allow for gene tree heterogeneity caused by a combination of ILS and high interlocus recombination but are potentially sensitive to both high intragenic and low intergenic recombination. We now examine studies of the effects of intra- and interlocus recombination on MSC-based species tree inference.

## Robustness of species tree inference

Species tree inference under the MSC assumes independence among loci (free interlocus recombination) and a single gene tree underlying each site within a locus (no intralocus recombination). These assumptions underlie both full likelihood species tree inference methods such as BPP (Flouri *et al.* 2020) and starBeast (Douglas *et al.* 2022) and summary statistical methods such as ASTRAL (Zhang *et al.* 2018). Under a coalescent model with recombination both assumptions may be violated. Here, we consider the findings of simulation studies of the coalescent with recombination that explore the effects of violations of these assumptions.

## Robustness to interlocus recombination

As noted by Zhu *et al.* (2022), nonindependence between loci (interlocus linkage disequilibrium) is expected to have less impact on inferences than intralocus recombination because assuming independence among jointly distributed random variables is a form of composite likelihood (CL) approximation and point estimates obtained using CLs are often at least statistically consistent. The greatest impact of using a CL is that it typically reduces the size of confidence intervals (or credible sets), causing overconfidence and coverage probabilities below the nominal values. Nevertheless, Wang and Liu (2016) conducted a simulation study that focused on the impact of using different bioinformatics methods to choose loci with higher intergenic recombination for analyses with ASTRAL II (Mirarab and Warnow 2015), which presumably might produce datasets with different levels of correlation between gene trees across loci. Two classes of methods were considered: methods based on linkage disequilibrium and methods based on breakpoint detection. They found that datasets generated using breakpoint detection methods tended to produce more accurate inferences of species tree topology. The underlying processes driving these observed performance patterns are not obvious from their study and more detailed studies are needed to identify causal relationships.

More recently, He *et al.* (2025) simulated gene trees under the coalescent with recombination and used these as input to ASTRAL to study the influence of correlations between gene trees (due to reduced interlocus recombination) on inferences of the species tree. They found that the accuracy of inferred species tree topologies (as measured by their Robinson–Foulds distance from the true tree) was reduced as recombination rates between loci decreased. However, this trend only persisted when fewer than about 1600 loci were used, suggesting that the inference method is statistically consistent in the face of lowered interlocus recombination rates. For smaller numbers of loci the variance is increased as one would expect for a CL method (if the observations have more statistical dependence their effective sample size is decreased). Because the authors did not simulate sequences to infer gene trees, instead treating gene trees as observations, this analysis ignores uncertainty due to gene tree inference and is therefore a best-case scenario for the ASTRAL method.

## Robustness to intralocus recombination

The effects of intralocus recombination are more difficult to predict and several studies have used simulations under the coalescent with recombination to examine their impact on MSC-based species tree inference methods (Lanier and Knowles 2012, Zhu *et al.* 2022, Yan *et al.* 2023). A small-scale study by Lanier and Knowles (2012) (using at most nine loci and three individuals per species) strongly suggested that inferences obtained using either the Bayesian MSC implementation in the starBeast program (Heled and Drummond 2010) or the approximate maximum-likelihood implementation in the STEM program (Kubatko *et al.* 2009) had little to no impact on inferences of the tree topology or divergence times. This finding was further confirmed in a large-scale simulation study by Zhu *et al.* (2022) who used up to 160 loci and eight sequences per species. Unlike the study of Lanier and Knowles (2012), which assumed complete genetic isolation among species, Zhu *et al.* (2022) also analysed data generated under an introgression model. They analysed the datasets using the BPP program either with introgression (MSC-I) or without (MSC). They simulated data with recombination rates centred around rate estimates for human and further including rates one order of magnitude above or below inferred values for human. Inferred species trees and divergence times were virtually unaffected by recombination. However, at the highest rates some positive bias was observed for estimates of θ and introgression times. We now consider the effects of intralocus recombination on estimates of θ and the underlying cause of these observed biases.

## Parameter inference with intralocus recombination

Schierup and Hein (2000) studied the effect of recombination on gene tree inference by simulating samples under the coalescent with recombination, inferring gene trees using either distance methods or maximum likelihood and calculating summary statistics that depend on tree shape but not mutation rate. They focused on examining the effects of recombination on tree shape because there is no one ‘correct’ gene tree in this case but instead a sequence of correlated gene trees associated with different sites. They found that ignoring recombination tended to produce coalescent events deeper in the inferred gene tree making the trees more star-like than expected under a neutral coalescent. This result has implications for population genetic inferences of parameters such as effective population size or population growth rate, but their simulation design (a single panmictic population) is not appropriate for studying the effects of recombination on inference of phylogenetic trees for genetically isolated species, and the MSC is the appropriate model for simulations in that case.

**Figure 2.**
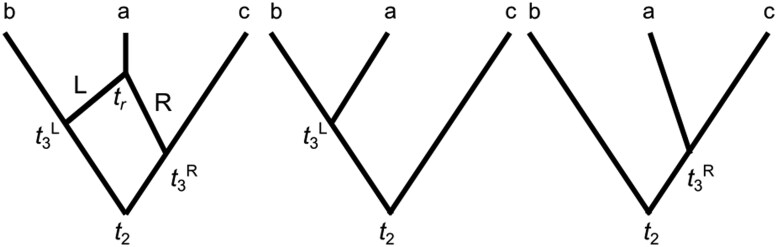
The ancestral recombination graph (ARG) of a sample of three sequences labelled a, b, and c with one recombination event involving sequence a at time $t_{r}$ shown at left. This ARG was used for analytical analysis and simulation studies of the effects of intralocus recombination. The gene tree in the middle of the figure (denoted tree L) is for the left half of the sequence generated by recombination and the tree on the right (denoted tree R) is for the right half of the sequence. The first coalescence event in tree L occurs at time $t_{3}^{L}$ and the first coalescence event in tree R occurs at time $t_{3}^{R}$. The final coalescence event at time $t_{2}$ is shared between the gene trees.

Here we further explore the influence of recombination on inferences of branch lengths in gene trees and the parameter θ within each population using a semi-analytical approach. Suppose that we sample three sequences (labelled *a*, *b*, and *c*) that experienced an intralocus recombination event creating the ARG shown in Figure 2. This produces two different gene tree topologies to the left and right of the recombination, hereafter referred to as the L and R topologies. The age of the root of the gene tree, denoted t2, is the same in the both trees L and R but the first coalescence time differs, where $t_{3}^{L}$ is the first coalescence time (between sequences *a* and *b*) in gene tree L and $t_{3}^{R}$ the first coalescence time (between *a* and *c*) in gene tree R. Assume that $n_{L}$ sites are sampled to the left of the recombination and $n_{R}$ sites are sampled to the right, where the sequence length for the locus is $n=n_{L}+n_{R}$. Sites mutate independently on each gene tree according to a Jukes–Cantor model (JC69). We focus on the expected site pattern frequencies which are sufficient statistics. A site pattern is a distinct combination of nucleotides among sampled sequences at a particular site. For example, ATT at a site would represent an A nucleotide in sequence *a*, and a T nucleotide in sequences *b* and *c*. For the JC69 model, the particular nucleotides are uninformative, only the patterns of sharing between sequences matter. With three sequences, there are five informative site patterns for estimating tree topology and branch lengths under this model, *xxx*, *xyz*, *yxx*, *xyx*, and *xxy*, where any sequences with the same letter at a site have the same nucleotides and sites are listed in the order *abc* with respect to the sequence in which they occur. For example, ATT and GCC both match site pattern *yxx*. The probabilities of patterns *xxx* and *xyz* do not depend on the tree topology, they depend only on the branch lengths, and nor does the probability of site pattern *yxx* differ for topologies L and R apart from the effects of differences in branch lengths. The probabilities for patterns *xyx* and *xxy* do differ between topologies L and R, but depend on only two underlying probabilities $P_{1}$ and $P_{2}$ that are swapped between patterns when the topology changes. The probabilities are presented in Table 1.

**Table 1.** Probabilities of site patterns on trees L and R of Figure 2.

	Site pattern				
Tree	*xxx*	*xyz*	*yxx*	*xyx*	*xxy*
L	$P(t_{3}^{L})$	$Q(t_{3}^{L})$	$P_{2}(t_{3}^{L})$	$P_{2}(t_{3}^{L})$	$P_{1}(t_{3}^{L})$
R	$P(t_{3}^{R})$	$Q(t_{3}^{R})$	$P_{2}(t_{3}^{R})$	$P_{1}(t_{3}^{R})$	$P_{2}(t_{3}^{R})$

The parameter $t_{2}$ is suppressed in our notation because it is a term that occurs in all probabilities. The formulas corresponding to these probabilities are given in equation 1.

For completeness, the site pattern probabilities are presented here, although without derivations—they are calculated using standard approaches for calculating likelihoods on trees (see Yang 2006) and simplifying the resulting formulas.


(1) $$\begin{array}{c} P(t_{3})=\frac{1}{16}\left(6e^{\frac{1}{3}(-4)(2t_{2}+t_{3})}+6e^{-\frac{8t_{2}}{3}}+3e^{-\frac{8t_{3}}{3}}+1\right),\\ Q(t_{3})=\frac{3}{8}\left(2e^{\frac{1}{3}(-4)(2t_{2}+t_{3})}-2e^{-\frac{8t_{2}}{3}}-e^{-\frac{8t_{3}}{3}}+1\right),\\ P_{1}(t_{3})=\frac{3}{16}\left(-2e^{\frac{1}{3}(-4)(2t1+t_{3})}-2e^{-\frac{8t_{2}}{3}}+3e^{-\frac{8t_{3}}{3}}+1\right),\\ P_{2}(t_{3})=\frac{3}{16}\left(-2e^{\frac{1}{3}(-4)(2t_{2}+t_{3})}+2e^{-\frac{8t_{2}}{3}}-e^{-\frac{8t_{3}}{3}}+1\right). \end{array}$$

The site patterns in a sample will follow a multinomial distribution with five categories corresponding to the counts of each of the five site patterns. The probabilities for each of the categorical variables are as specified in Table 1. We define the observed site pattern counts as


$$n=\{n_{\mathrm{xxx}},n_{\mathrm{xyz}},n_{\mathrm{yxx}},n_{\mathrm{xyx}},n_{\mathrm{xxy}}\}.$$

The log likelihoods of the data under tree topologies L and R are


(2) $$\begin{array}{c} \ell_{L}(t_{2},t_{3}^{L}|n)=\text{log}\;C+n_{\mathrm{xxx}}\;\text{log}\;P(t_{3}^{L})\\ +n_{\mathrm{xyz}}\;\text{log}\;Q(t_{3}^{L})+n_{\mathrm{yxx}}\;\text{log}\;P_{2}(t_{3}^{L})\\ +n_{\mathrm{xyx}}\;\text{log}\;P_{2}(t_{3}^{L})+n_{\mathrm{xxy}}\;\text{log}\;P_{1}(t_{3}^{L}), \end{array}$$

(3) $$\begin{array}{c} \ell_{R}(t_{2},t_{3}^{R}|n)=\text{log}\;C+n_{\mathrm{xxx}}\;\text{log}\;P(t_{3}^{R})\\ +n_{\mathrm{xyz}}\;\text{log}\;Q(t_{3}^{R})+n_{\mathrm{yxx}}\;\text{log}\;P_{2}(t_{3}^{R})\\ +n_{\mathrm{xyx}}\;\text{log}\;P_{1}(t_{3}^{R})+n_{\mathrm{xxy}}\;\text{log}\;P_{2}(t_{3}^{R}), \end{array}$$

where *C* is a constant that does not depend on the coalescent times. Equations 2 and 3 can be numerically maximized to jointly estimate $t_{2}$ and $t_{3}$. If we are instead interested in estimating θ for the sites associated with topology L or R we can marginalize over the coalescent process using numerical integration as follows:


(4) $$L(n|\theta ,T)=\int_{0}^{\infty }\int_{0}^{t_{2}}L(n|t_{2},t_{3})f(t_{2},t_{3}|\theta ,T)dt_{3}dt_{2},$$

where L is the likelihood under either topology L or R and the joint density of the coaescent times is


$$f\left(t_{2},t_{3}\right)=\frac{6}{\theta }e^{-\frac{6}{\theta }t_{3}}\frac{2}{\theta }e^{-\frac{2}{\theta }(t_{2}-t_{3})}.$$

We conducted two small simulation studies to examine the effects of recombination (in this particular example) on inferences of either coalescence times on gene trees or θ. Although we are usually not interested in inferring gene tree coalescence times, these are the source of information about θ and, as we shall see, biases in coalescent time inferences help explain the bias observed in inferences of θ.

## Inference of θ with recombination

We simulated coalescence times for 500 samples each of n=200 sites ($n_{L}=100$ and $n_{R}=100$) conditional on the ARG with one recombination resulting in the L and R trees shown in Figure 2. We used a relatively large value of θ=0.1 and a per-site recombination rate of $r=10^{-5}$. We conducted maximum-likelihood inference on three subsets of data by numerically integrating equation 4 using the NIntegrate function in Mathematica v.12 to infer the likelihood and then maximizing the logarithm of this likelihood as a function of θ using the FindMaximum function in Mathematica. The three subsets of data (and likelihood functions used) were as follows: (i) the entire sequence of n=200 sites using the likelihood function for topology L; (ii) the $n_{L}=100$ sites to the left of the recombination using the likelihood function for topology L; and (iii) the $n_{R}=100$ sites to the right of the recombination using the likelihood function for topology R. In this case, we are conditioning on one particular recombination scenario, considering a relatively small number of sites and only a single locus so we expect estimates of θ to be imprecise and potentially biased. However, the main interest is in the differences of average inferences and bias between the analysis of the complete sequence dataset (ignoring recombination and incorrectly using a likelihood function for the L topology) and the two analyses using a correct topology on nonrecombinant subsequences to the left and right of the recombination. The mean of the maximum-likelihood estimates (MLEs) across the 500 simulated datasets [and 95% confidence interval (CI)] are ${\bar{\theta }}_{L}=0.15\pm 0.01$, ${\bar{\theta }}_{R}=0.15\pm 0.01$, and ${\bar{\theta }}_{M}=0.18\pm 0.01$, where subscripts L, R, and M denote the analyses for the left, right, and combined sequences, respectively. Thus, there is positive systematic bias for all the MLEs but the impact of recombination is to further inflate the bias in estimates of θ as has been observed in previous simulation studies.

## Inference of coalescence times with recombination

We simulated coalescence times for 500 samples each of n=100000 sites. Coalescent times were measured in units of expected substitutions per site. MLEs were obtained by jointly maximizing the log-likelihood functions of equations 2 and 3 using the FindMaximum function in Mathematica. The MLEs of coalescent times for sequences to the left and right of the recombination were essentially perfect estimates of the true values with this sample size. We focus on investigating the systematic bias for MLEs of the coalescent times of recombinant sequences obtained by maximizing the log-likelihood for topology L. We calculated two summary statistics. Since the coalescence time $t_{2}$ is a common parameter for both topologies L and R we examined the average bias defined as:

 . $$B_{2}=\frac{1}{n}\sum_{i=1}^{n}\left({\hat{t}}_{2}-t_{2}\right).$$

In comparing the estimates of $t_{3}$ we compared the MLE with the average of true value of $t_{3}$ on each topology:


$$B_{3}=\frac{1}{n}\sum_{i=1}^{n}\left({\hat{t}}_{3}-\left[\frac{t_{3}^{L}+t_{3}^{R}}{2}\right]\right).$$

In the simulations, the average coalescent times were ${\bar{t}}_{2}=0.0820$, ${\bar{t}}_{3}^{L}=0.0268$, and ${\bar{t}}_{3}^{R}=0.0261$. The bias values were $B_{2}=-0.0158$ and $B_{3}=0.0249$. In particular, estimates of $t_{3}$ were virtually always overestimates and estimates of $t_{2}$ were virtually always underestimates, making the trees more starlike; this accounts for the overestimates of θ.

## ACCOMMODATING RECOMBINATION

Approaches aimed at accommodating recombination in phylogenetic analyses are based on the assumption that recombination biases results of phylogenetic analyses and therefore needs to be explicitly considered, either by removing recombinant loci or by using methods that aim to allow for inter- or intralocus recombination. Such methods are considered here.

## Recombination detection

One class of methods whose goal is to accommodate recombination in phylogenetic analyses aim to detect intralocus recombination and exclude putative recombinant loci from subsequent analyses (Hey and Nielsen 2004, Potter and Jennings 2023). Recombination inference methods have also been used to choose loci at sufficiently large intervals to insure high levels of interlocus recombination (as in Wang and Liu 2016). Based on findings of simulation studies it seems that intralocus recombination may have little impact on the results (species tree topology and divergence times) of species tree inference under the MSC (Lanier and Knowles 2012, Zhu *et al.* 2022) so it may be unnecessary to preprocess the data to remove recombinant sequences. Moreover, the expected amount of recombination at a locus is a function of the age of the sampled sequences at that locus; more distantly related sequences at a locus will have experienced more recombination on average (Hey and Nielsen 2004). Thus, removing loci with recombinant sequences has the potential to bias parameter estimates because it may preferentially remove loci at which sequences have older coalescent times, influencing estimates of θ. Also, as noted below, some recombination events will not be detectable using sequence data. Simulation studies are needed to evaluate the effects of recombination detection and exclusion methods on inferences. Nonetheless, recombination detection strategies have been used extensively in the published literature and so we briefly review methods for inferring recombination and detecting recombination breakpoints here.

**Figure 3.**
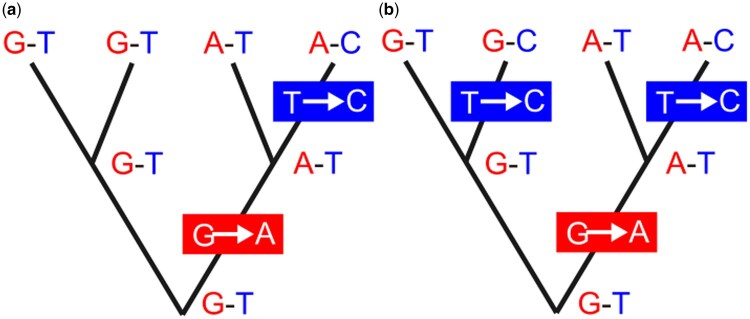
The basic principle underlying the four gamete test is illustrated for a sample of SNPs at two linked loci. The allele at the first locus on the chromosome is indicated in red and the allele at the second locus in blue. Mutations are indicated in a box where the colour (blue or red) indicates the locus that mutates. The box is positioned on the gene tree lineage where the mutation occurs. Ancestral haplotypes at each of the three coalescent events on each gene tree are shown as well as the four sampled haplotypes at the tips of each gene tree (top). The gene tree of panel A shows a case with three distinct haplotypes (G-T, A-T, and A-C) in which a single mutation at each locus can explain the observed haplotypes (e.g. one red box and one blue box). The gene tree of panel B shows a case with four distinct haplotypes (G-T, G-C, A-T, and A-C) that cannot be explained without at least two mutations at some locus (in this case at the second locus, one red box and two blue boxes). Assuming an infinite sites model in which no more than one mutation occurs per SNP, a recombination is required to account for the four haplotypes in B, for example a recombination between haplotypes G-T and A-C.

One of the earliest recombination detection methods is the so-called four gamete test (Hudson and Kaplan 1985). This test is based on the observation that under an infinite sites model in which no site has more than one mutation on a gene tree it is impossible to observe all four possible haplotypes at a pair of linked loci unless at least one recombination occurs. Thus, if four gametes are observed at a pair of linked biallelic loci recombinations must have occurred. However, this places a lower bound on the number of recombinant loci because recombinations can occur that do not produce all four haplotypes (Hudson and Kaplan 1985). It is also possible that the infinite sites assumption is violated and recurrent mutations occurred which can also lead to four haplotypes. Despite these drawbacks the method provides a simple way to flag loci that are likely to have undergone recombination and continues to be used in many studies (Fig. 3).

Numerous recombination detection methods have been developed over the last several decades using a variety of approaches and sources of information. One class of methods are similar to the four gamete test in that they rely on an assumption of low mutation rate relative to recombination rate and use principles of compatibility analysis or parsimony (with homoplasies indicating recombination) (Stephens 1985, Sawyer 1989, Maynard Smith 1992, Maynard Smith and Smith 2002, Bruen *et al.* 2006). Another related class of methods aim to detect changes of gene trees along a sequence. Most of these methods involve some form of breakpoint detection (see section on modelling recombination change points) signalling a change in gene tree topology and/or branch lengths due to recombination. Some of these methods are similar to the likelihood ratio test (LRT) of topology discussed below (section on phylogenetic mixture models) (Huelsenbeck and Bull 1996) but consider a sliding window of likelihoods along a sequence to detect change-points (Grassly and Holmes 1997, Kosakovsky Pond *et al.* 2006b). Others focus on detecting a change in the inferred gene tree, either through inference of gene trees in a sliding window and calculating distances between inferred trees on different intervals (McGuire *et al.* 1997), or by comparing bootstrap proportions (Salminen *et al.* 1995) or Bayesian posterior probabilities (Paraskevis *et al.* 2005) or via a hidden Markov model (HMM) in which gene trees can change between adjacent sites (see section on modelling recombination change points). A potential weakness of the topology change detection methods is that many recombinations do not produce a change in the local gene tree topology (Hein *et al.* 2004) which will reduce the power of the methods to detect recombination.

Yet another class of recombination detection methods rely on population genetics models; these models are explicitly based on expectations for a single panmictic population and are not appropriate for use with structured populations or sequences from different species (Tsaousis *et al.* 2005). Some methods use permutation tests based on summary statistics of linkage disequilibrium (Miyashita and Langley 1988, Schaeffer and Miller 1993) while others are based on CL approximations to the ARG (Martin *et al.* 2010). Although it is possible to infer recombination rates using a full likelihood approach by integrating over the ARG using MCMC (Kuhner *et al.* 2000, Nielsen 2000, Wang and Rannala 2008) it is computationally intensive and the methods do not usually attempt to infer whether particular subregions of loci are likely to be recombinant, instead being focused on estimating recombination rates on intervals. Finally, much recent interest has focused on inferring the ARG itself for a population sample from a single panmictic population (reviewed in Nielsen *et al.* 2025). Although, in principle, the ARG contains a history of recombinations on particular intervals of the genome it is typically poorly estimated with much statistical uncertainty (one reason that earlier methods integrated over the ARG). The ARG inference approach is often applied to detect selection, although the model is based on a neutral coalescent. In summary, population genetic methods are currently only suitable for detecting recombination among neutral sites within single isolated populations and are unsuitable for use in most species tree inference problems.

## Phylogenetic accommodation of recombination

Another approach to accommodating recombination is to develop phylogenetic inference methods that explicitly take account of gene tree variation across genome sequences. At least three approaches exist: modelling sites as conditionally independent, with the gene tree underlying a site drawn from a distribution of two or more gene trees that are jointly inferred (mixture models); modelling change points along the genome at which the gene tree underlying the sequence data changes and thus the sequence likelihood changes (change-point models); and using a phylogenetic inference model based on independent single nucleotide polymorphisms (SNPs) which each have a different underlying gene tree—such a method is insensitive to intralocus recombination, although still assuming independence among SNPs (high levels of inter-SNP recombination). Here we explore examples of each approach.

## Phylogenetic mixture models

Recognizing that recombination and ancestral polymorphism can create conflicts between a gene tree and a species tree, Huelsenbeck and Bull (1996) proposed an LRT for a single gene tree versus a mixture of gene trees. The LRT assumes that data partitions of the sites are specified, limiting its utility for detecting recombination because recombination breakpoints associated with changes of the gene tree will typically be unobserved random variables. Ané *et al.* (2007) proposed a related Bayesian inference method that allows each gene/locus to have a potentially distinct gene tree topology and a substitution process with independent parameters. They use a Dirichlet process prior on topologies, which is a generalization of the multinomial distribution that allows an unknown number of categories (in this case, each distinct topology is a category). As in the Huelsenbeck and Bull (1996) method, it is assumed that the partitions are known (in this case they are the genes/loci) and only one topology underlies each partition. The joint posterior distribution of gene trees for each partition is estimated and in a second stage analysis used to infer the posterior density of concordance factors for each clade.

Both Boussau *et al.* (2009) and Wong *et al.* (2024) proposed a mixture model similar to the theoretical mixture models described previously that assumes sites are exchangeable and conditionally independent. Individual sites are assigned to partitions that allow different gene trees. The methods provide probabilities for gene trees and potentially accommodate intralocus recombination but do not directly infer a species tree and are impractical for use with more than a handful of sequences. These approaches can allow an estimate of a common phylogeny but do not specify an explicit model in doing so. Presumably the methods will perform best in cases where the most common gene tree (or clade) is the true gene tree; this would not be the case, for example, when sampling gene trees in the so-called anomaly zone (Degnan and Rosenberg 2006), defined by a combination of MSC parameters for which the expected most frequent gene tree does not match the species tree. Moreover, the gene partitions in the method of Ané *et al.* (2007) must be prespecified and are unlikely to match the recombination breakpoints between distinct topologies.

## Modeling recombination change points

Another approach to accommodate recombination that is related to mixture models uses a change-point model to identify positions in a genome sequence at which the gene tree topology changes (presumably due to recombination). This approach has the advantage over simple mixture models (described above) in that it does not require the regions with a common topology to be prespecified, identifying change-points as part of the inference process. It also has the advantage over site-based mixture models that it places more probability on physically adjacent sites having a shared gene tree, which is more likely under a gene conversion or crossing-over process. However, under a coalescent with recombination physically disjunct sequence tracts can have a shared gene tree (the recombination process ALONG a chromosome is not Markovian) and this possibility is not adequately modelled under the existing change-point models. Most change-point (or breakpoint) models use a heuristic HMM to predict changes of topology along a sequence (McGuire *et al.* 2000, Suchard *et al.* 2002, Minin *et al.* 2007, Kosakovsky Pond *et al.* 2006a, Boussau *et al.* 2009, Westesson and Holmes 2009, Didelot and Wilson 2015). However, existing methods are computationally expensive and do not provide a direct way to infer a species tree using the samples of gene trees inferred for each identified sequence tract with a common gene tree.

## SNP-based MSC inference methods

Another possible solution to the potential problems caused by intralocus recombination is to restrict analyses to a single polymorphic site per locus. In this case, intralocus recombination is irrelevant and the state space is simple enough that one can analytically integrate over gene tree uncertainties at each locus, rather than using MCMC as is done in multisite sequence analysis. Bryant *et al.* (2012) implemented a Bayesian species tree inference method using individual SNPs that are assumed to be independent (e.g. high levels of inter-SNP recombination). The tradeoff with this approach is that there is a large loss of information due to the use of only a single site which carries little information about the gene tree underlying the SNP.

## DISCUSSION

The effects of recombination on phylogenetic inference have been a subject of study by both phylogeneticists and population geneticists for many decades. We have reviewed a range of different approaches to the problem: using simulation and analytical theory to study the robustness of gene tree inference (concatenation analysis) and species tree inference methods to recombination; using computational methods to detect (and potentially eliminate) recombinant loci; and using either breakpoint detection or mixture models to allow gene trees to vary across the genome. Results from analyses of statistical robustness suggest that many species tree inference methods are little affected by recombination when the interest is in species divergence times and species tree topology. However, intralocus recombination may cause small but significant biases in parameters such as effective population sizes (θ) and inrogression proportions in structured populations or hybridizing species. Concatenation analysis of species trees, on the other hand, can be seriously misled by chimeric sequences created by recombination across the genome unless most underlying gene trees match the species tree; this is expected only under special circumstances. Similar to the effect of intralocus recombination on gene trees, making them more starlike, we expect branches on phylogenies produced by concatenation to also become more starlike when recombination and ILS occur, compressing divergence times between nodes. Although recombination detection has many useful applications, detecting and removing recombinant sequences is probably unnecessary for species tree inference and may lead to biases in estimates of species divergence times and other demographic parameters. In any case, recombination detection methods based on gene tree topology changes along a sequence are more appropriate for use in species tree inference than those based on population genetic methods (which assume a single panmictic population).

A wide range of methods exist for inferring gene trees underlying different regions of a genome (or different sites of a sequence). However, these methods are typically very computationally intensive and do not provide a model-based procedure for combining inferences from gene trees to infer a species tree, typically using heuristics such as assuming that the most common gene tree topology, the most frequent bipartition, or the highest marginal posterior probability for a clade represents the most probable species tree relationship. A method extending MSC-based species tree inference to include ARGs within species would lead to the appropriate statistical weights for gene trees on intervals but is very difficult to efficiently implement in a computer program and has so far not been developed.

## Data Availability

There are no data associated with this manuscript.
